# Using Synchrotron Radiation Imaging Techniques to Elucidate the Actions of Hexarelin in the Heart of Small Animal Models

**DOI:** 10.3389/fphys.2021.766818

**Published:** 2022-01-21

**Authors:** Mark T. Waddingham, Hirotsugu Tsuchimochi, Takashi Sonobe, Ryotaro Asano, Huiling Jin, Connie P. C. Ow, Daryl O. Schwenke, Rajesh Katare, Kohki Aoyama, Keiji Umetani, Masato Hoshino, Kentaro Uesugi, Mikiyasu Shirai, Takeshi Ogo, James T. Pearson

**Affiliations:** ^1^Department of Advanced Medical Research for Pulmonary Hypertension, National Cerebral and Cardiovascular Center, Suita, Japan; ^2^Department of Cardiac Physiology, National Cerebral and Cardiovascular Center Research Institute, Suita, Japan; ^3^Department of Physiology, School of Biomedical Sciences, Heart Otago, University of Otago, Dunedin, New Zealand; ^4^Japan Synchrotron Radiation Research Institute, Harima, Japan; ^5^Department of Physiology, Monash Biomedicine Discovery Institute, Monash University, Clayton, VIC, Australia

**Keywords:** microangiography, X-ray diffraction, myosin cross-bridges, pulmonary hypertension, endothelial dysfunction, microvessels, cardiomyocytes

## Abstract

The majority of the conventional techniques that are utilized for investigating the pathogenesis of cardiovascular disease in preclinical animal models do not permit microlevel assessment of *in situ* cardiomyocyte and microvascular functions. Therefore, it has been difficult to establish whether cardiac dysfunction in complex multiorgan disease states, such as heart failure with preserved ejection fraction and pulmonary hypertension, have their origins in microvascular dysfunction or rather in the cardiomyocyte. Herein, we describe our approach of utilizing synchrotron radiation microangiography to, first, ascertain whether the growth hormone secretagogue (GHS) hexarelin is a vasodilator in the coronary circulation of normal and anesthetized Sprague-Dawley rats, and next investigate if hexarelin is able to prevent the pathogenesis of right ventricle (RV) dysfunction in pulmonary hypertension in the sugen chronic hypoxia model rat. We show that acute hexarelin administration evokes coronary microvascular dilation through GHS-receptor 1a and nitric oxide, and through endothelium-derived hyperpolarization. Previous work indicated that chronic exogenous administration of ghrelin largely prevented the pathogenesis of pulmonary hypertension in chronic hypoxia and in monocrotaline models. Unexpectedly, chronic hexarelin administration prior to sugen chronic hypoxia did not prevent RV hypertrophy or RV cardiomyocyte relaxation impairment. Small-angle X-ray scattering revealed that super relaxed myosin filaments contributed to diastolic dysfunction, and that length-dependent activation might contribute to sustained contractility of the RV. Thus, synchrotron-based imaging approaches can reveal novel insights into cardiac and coronary functions *in vivo*.

## Introduction

The origins of cardiovascular disease have often been thought of as either being in the vasculature or in the myocardium, but more often than not, the techniques employed to investigate the origins of pathophysiological states that affect multiple organs are not sensitive enough to detect early changes in structure and in function in preclinical animal models. Herein, we propose that techniques utilizing synchrotron radiation (SR)-based high-temporal and -spatial resolution imaging, and X-ray diffraction *in vivo* provide an integrative approach to investigating vascular and myocardial roles in the pathophysiology of heart failure in small animal models on the micro- and nanoscales and are readily applied in combination with gold-standard cardiovascular techniques. Furthermore, these techniques have great potential to investigate the efficacy of potential pharmacological therapies and other interventions for heart failure. In this brief report on bridging techniques, we outline the application of these protocols in ongoing studies that investigate the suggested cardioprotection actions of the synthetic peptide hexarelin. For detailed descriptions of the first principles of these SR techniques, their validation, limitations, and strengths, the reader is referred to our earlier studies (Pearson et al., 2007; Schwenke et al., 2007; Shirai et al., 2009, 2013; Jenkins et al., 2012, 2013). First, we briefly describe our motivation for directly assessing the physiological actions of this ghrelin analog *in vivo*.

Pulmonary hypertension (PH) is a rare disease with poor long-term prognosis, and can have either genetic or environmental origins, and in one classification group also has its origins in LV dysfunction or valvular disease. Various known factors that compromise pulmonary endothelial function also lead to progressive remodeling of the pulmonary arteries and/or veins, and together, leads to elevated pulmonary arterial pressure (>25 mmHg at rest), as well as increased afterload in the right ventricle (RV) (Thenappan et al., 2018). Initially, the increased work imposed on the RV to maintain right heart output stimulates RV hypertrophy (adaptive phase) but becomes maladaptive with RV dilatation, increasing myocardial energetic demand, thereby, resulting in greatly elevated risk of death (Vonk Noordegraaf et al., 2017; Sharifi Kia et al., 2021). Activation of the sympathetic nervous system, endothelin-1, and adrenal production of catecholamines is considered to be important both for the early compensatory increase in RV contractility and in subsequent RV dysfunction and remodeling in the long term (De Man et al., 2013; Vaillancourt et al., 2017; Cassady and Ramani, 2020; Prisco et al., 2020). Recently, some of us demonstrated in two experimental models of PH (monocrotaline and Sugen/chronic hypoxia, SuHx) that the impairment of vascular responses in the right coronary artery (RCA) is also associated with elevated RV afterload and contributes to the early progression of heart failure in PH (Inagaki et al., 2021). Both pulmonary artery denervation and renal sympathetic denervation are being considered as potential strategies to reduce sustained neurohormonal activation in PH alongside pharmacological therapies (Vaillancourt et al., 2017).

After more than 20 years since the discovery of the growth hormone secretagogue (GHS) ghrelin (Kojima et al., 1999), there is still great interest in the cardiovascular actions of this small peptide-mediated principally through the known G-protein-coupled receptor, which is the GHS receptor 1a (GHS-R1a). Although ghrelin is essentially produced by the gut, GHS-R1a is widely expressed in the heart and vasculature, and has been shown to mediate most of ghrelin's physiological actions in these systems, independent of growth hormone release. Various studies by our group and many other laboratories have shown that chronic administration of ghrelin in humans and rodent models appreciably improves left ventricle (LV) function in both acute and chronic heart failures (Nagaya et al., 2004; Schwenke et al., 2008a; Soeki et al., 2008; Mao et al., 2013b, 2015; Mcdonald et al., 2020), improves endothelial function (Shimizu et al., 2003; Virdis et al., 2015; Pearson et al., 2018), and also ameliorates both inflammatory pulmonary injury (Wu et al., 2007; Sukumaran et al., 2018) and the early pathogenesis of pulmonary hypertension (Henriques-Coelho et al., 2004; Schwenke et al., 2008b, 2011). Notably, both one bolus and chronic administrations of ghrelin have been shown to ameliorate acute and chronic LV dysfunction and fibrotic remodeling of post myocardial infarction through potent sympathoinhibition and vagal activation (Schwenke et al., 2008a; Soeki et al., 2008; Mao et al., 2013b; Shirai et al., 2015).

Despite the clear benefits of ghrelin as a therapy to ameliorate myocardial dysfunction and adverse cardiac remodeling in hypertension, as well as both acute and chronic heart failure, the two important limitations hindering it as a viable therapy are: that ghrelin peptide is currently expensive to produce clinical therapeutic doses and that it has a short half-life (9–13 min). For more than a decade, there has been a great interest in GHS alternatives to ghrelin as a therapy. Hexarelin is a stable synthetic GHS peptide analog of ghrelin that has been shown to have a wide range of cytoprotective and cardioprotective actions *in vitro* and *in vivo*, such as amelioration of LV dysfunction and remodeling in the setting of pressure overload and myocardial infarction (Berti et al., 1998; Filigheddu et al., 2001; Mao et al., 2013a, 2014a,b; Mcdonald et al., 2020). Hexarelin was demonstrated to have greater potency than ghrelin treatment in improving LV function after myocardial infarction in ghrelin deficient mice, which was suggested to be due to the possible binding of hexarelin to cardiac GHS-R1a and CD36 (class B scavenger receptor involved in fatty acid uptake) receptors (Mao et al., 2013a). Nevertheless, others have suggested that CD36 mediates vasoconstriction in the coronary circulation, as well as negative inotropic and chronotropic responses in *ex vivo* mouse hearts (Bodart et al., 2002). Both ghrelin and hexarelin are considered to exert comparable effects on the improvement of cardiac autonomic balance post myocardial infarction (Mao et al., 2014b).

For the purpose of demonstrating our approach to bridge the limitations of conventional techniques in assessing coronary microcirculation and myocardial function *in vivo* utilizing SR imaging techniques, we report our initial findings that investigated the acute and chronic effects of hexarelin administration in rats. In study 1, we tested the hypothesis that acute administration of hexarelin induces coronary vasodilation in normal Sprague-Dawley rats utilizing SR microangiography. In parallel independent studies 2 and 3, we tested the hypothesis that chronic pretreatment with hexarelin is cardioprotective and prevents RV dysfunction in the SuHx rat model of severe PH with RV failure (Abe et al., 2010) by SR small-angle X-ray scattering (SAXS) to investigate *in vivo* sarcomeric function, and by microangiography to assess RV perfusion.

**Figure 1:**
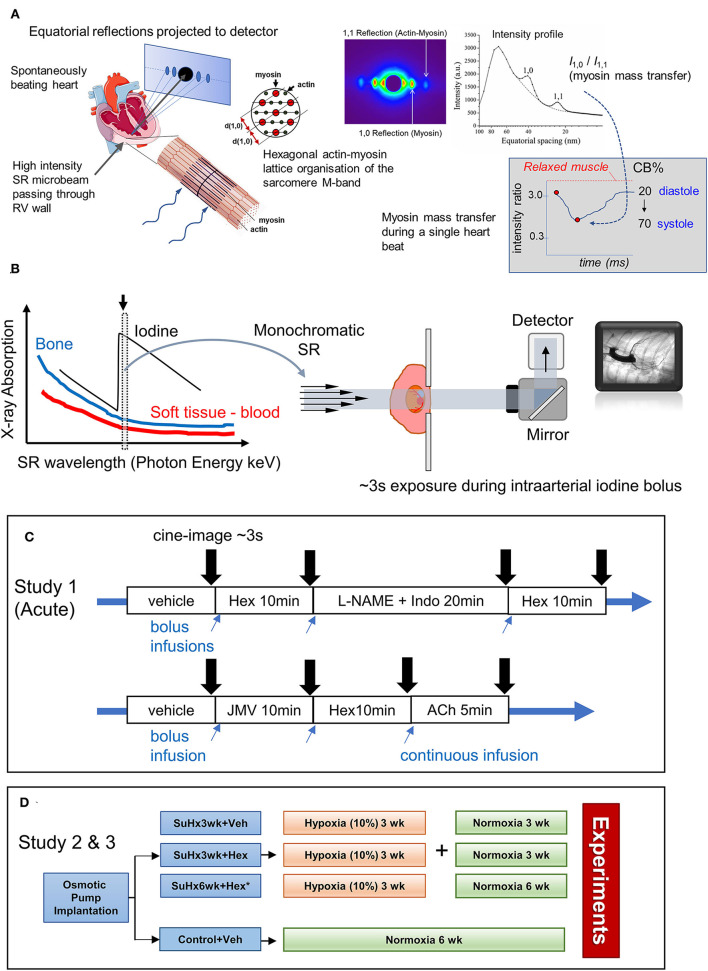
Schematics illustrating the application of synchrotron radiation (SR)-based small-angle X-ray scattering (SAXS) to *in situ* beating hearts of anesthetized rodents, SR microangiography of the coronary circulation and protocols in studies 1–3. **(A)** Equatorial X-ray reflections are continuously recorded as scatter about the central path of the X-ray microbeam from perpendicularly aligned cardiac muscle fibers within localized regions of the left or right ventricle (RV) because of the crystal-like hexagonal lattice organization of the two predominant myofilament proteins, actin and myosin. Each SAXS pattern from a 10–15 ms exposure produces sufficient reflection intensity to estimate myosin mass transfer to actin filaments (intensity ratio of 1,0 reflection to 1,1 reflection) at any given point in the cardiac cycle. From SAXS patterns recorded over ~3s the changes in electron distribution within the actin-myosin lattice during the cardiac cycle are utilized to inform on myosin-head proximity to actin filaments and force-producing cross-bridge (CB) formation in systole (following calibration against relaxed muscle and rigor muscle states of no CB attachments and 100% attachment, respectively). Furthermore, the interfilament spacing (d_1,0_) that can also be derived from each SAXS pattern is related to the sarcomere length of the cardiac fibers in that myocardial layer. **(B)** SR microangiography enables optimization of visualization of coronary microvessels of anesthetized rodents. Several orders of magnitude brighter photon intensity of SR and high degree of collimation relative to conventional X-ray sources facilitate both fast and highly spatially resolved absorption imaging (Shirai et al., 2013). Tuning X-ray energy to a narrow bandwidth just above the K-edge of iodine contrast agent maximizes absorption contrast. In this study to achieve a view of the left coronary circulation, rats are placed in a supine position (lateral view) while a frontal view of the right coronary circulation is achieved by suspending the animals on their side in the path of the beam. **(C)** Two protocols in study 1 for repeated imaging of the same anesthetized rats during hexarelin bolus administration before and after blockade of nitric oxide and prostanoids (L-NAME and indomethacin treatment) or alternatively hexarelin after pretreatment with GHS-R1a inhibitor (JMV2959). Times indicate the period of equilibration prior to imaging. **(D)** Group allocation and timeline for studies 2 (microangiography) and 3 (SAXS) performed on independent rats. Further details are provided in the Methods text.

SR microangiography permits the highest temporal and spatial resolution 2D imaging of mouse and rat coronary arterial vessels *in vivo* at physiological heart rates during anesthesia, facilitating investigations of endothelial and smooth muscle functions across the macro- and microvessel networks simultaneously during pharmacological stimulation (Shirai et al., 2009, 2013). On the other hand, SAXS has long been performed to investigate actin-myosin cross-bridge regulation, and thick and thin filament activations in isolated cardiac muscle (Irving et al., 2000; Konhilas et al., 2002; Yagi et al., 2004; Colson et al., 2007). In recent years, we have applied fast SR-based SAXS to study *in vivo* cross-bridge dynamics in the exposed intact heart of anesthetized rodents, albeit in the intact heart, only equatorial reflections can be recorded (Figure 1A). To date, we have shown in prediabetic and diabetic rats that this approach is sensitive enough to detect impaired relaxation of *in situ* cardiomyocytes, regional differences in impairment across the LV free wall, and changes in myosin thick filament activation that correlated with altered myofilament phosphorylation and protein kinase activity (Jenkins et al., 2013; Waddingham et al., 2015, 2019). Furthermore, we suggest that myosin interfilament spacing (d_1,0_ spacing) can be performed to estimate sarcomere length and sarcomere shortening in a local region based on established relationships between sarcomere length of intact cardiac muscle and d_1,0_ spacing (Yagi et al., 2004; Toh et al., 2006; Waddingham et al., 2019), providing an important index of local work and biomechanical function as well as pathological changes, for example, contracture or overstretching.

## Materials and Methods

### Ethics Statement

All experiments involving animals were conducted in accordance with guidelines of Physiological Society of Japan and approved by the animal experimentation committee at the National Cerebral and Cardiovascular Center (Proposal Nos. 18077, 19013, 19053, 20010, and 21048), and the Japan Synchrotron Radiation Research Institute (SPring-8 Proposals 2017A1470, 2018A1193, 2018A1242, 2018B1086, 2018B1190, and 2019A1077). The reporting of animal studies in this article complies with the ARRIVE guidelines for animal research.

### Animals: Acute Study 1

Ten-week old male Sprague-Dawley rats (*n* = 10) were sourced from Japan SLC (Kyoto, Japan) for preliminary investigation of the coronary dilator mechanisms in response to acute administration of hexarelin in the healthy coronary circulation of rats during terminal experimentation under anesthesia as described below.

### Animals and Study Design: Study 2 (Microangiography/Hemodynamics) and Study 3 (SAXS)

Seven-week old male Sprague-Dawley rats (*n* = 50) were sourced from Japan SLC (Kyoto, Japan). The rats were randomized to the control vehicle (*n* = 7, study 2; *n* = 8, study 3), sugen/chronic hypoxia (SuHx) vehicle (*n* = 7, studies 2 and 3), and hexarelin-treated SuHx (*n* = 7, studies 2 and 3) groups (see Figure 1D for group allocations). In order to maintain the continuity of a chronic hypoxia environment, subcutaneously implanted osmotic pumps were chosen for the administration of hexarelin or vehicle prior to chronic hypoxia exposure. Both hexarelin (100 μg·kg^−1^·day^−1^, equivalent to a ghrelin dose of 400 μg·kg^−1^·day^−1^) and vehicle (0.9% saline) were delivered with osmotic minipumps (model 2ML4, Alzet® Durect Corporation, Cupertino, CA, United States) for 4 weeks and by daily subcutaneous injections for the final 2 weeks (all the animals in a normoxic environment). Under light anesthesia (isoflurane 1.5%, mixed with room air; Pfizer, Tokyo, Japan), free-breathing rats were placed in a prone position, and using an aseptic technique, a small incision was made between the scapulae. By blunt dissection, a path was made under the skin to facilitate adequate insertion depth of the osmotic minipump. The incision was closed using a 4–0 suture with an interrupted suture pattern. The rats were administered a pain reliever (carprofen 5 mg·kg^−1^s.c.; Zoetis, Tokyo, Japan) and then allowed to recover. Once adequate recovery was confirmed (24–48 h post minipump implantation) rats in the SuHx groups received a single injection of sugen 5416 (20 mg·kg^−1^, s.c.; MedChemExpress, New Jersey, USA) (Abe et al., 2010) dissolved in a 0.5% CMC buffer (4 ml/kg) and then were immediately housed in a hypoxia chamber (10% O_2_ by nitrogen mixing) for 3 weeks. Subsequently, the SuHx rats were maintained under normoxic (room air) conditions for a further 3 weeks (termed SuHx3wk). The control rats received an equivalent bolus volume (4 ml·kg^−1^) of 0.5% CMC solution and were maintained under normoxic conditions for the 6-week duration of the study. In study 3, we also included a group of SuHx rats that were treated with hexarelin for an additional 3 weeks (SuHx6wk).

### Microangiography System, and Hemodynamic Evaluation and Imaging Protocols: Studies 1 and 2

The coronary circulation of anesthetized rats (1.5–2% isoflurane) was visualized by monochromatic SR tuned to 34 keV (K-edge of iodine) (Figure 1B) at public beamline BL20B2, and 33.2 keV at BL28B2 (SPring-8 Synchrotron, Hyogo, Japan), as previously described (Jenkins et al., 2012; Waddingham et al., 2019; Inagaki et al., 2021). The rats were placed in front of the X-ray detector (C11440 Orca Flash 4.0; Hamamatsu Photonics, Hamamatsu, Japan) with a 200-μm GAGG scintillator in the path of the X-ray beam with a field of view of 15 mm (horizontal) × 7 mm (vertical) (pixel size 13 μm) in study 1, and 31 × 24.5 mm (pixel size 15.3 μm) in study 2. In study 1, the rats were placed in a supine position for a lateral view of the left coronary artery. In protocol 1 (Figure 1C), following baseline imaging, repeated images were acquired: (1) 10 min after the administration of hexarelin (100 μg·kg^−1^i.v.), (2) 20 min after combined nitric oxide synthase and cyclooxygenase inhibition (L-NAME 50 mg·kg^−1^ i.v. and indomethacin 5 mg·kg^−1^; Sigma-Aldrich, Tokyo, Japan), and (3) and repeat administration of hexarelin. In protocol 2, using separate animals, the response to hexarelin was examined after GHS-R1a blockade (JMV2959 5 mg·kg^−1^ i.p.; MedChem Express) followed by acetylcholine (ACh 5 μg·kg^−1^·min^−1^) i.v. infusion to demonstrate that endothelium-dependent dilation was not impaired. In study 2, the rats were positioned to facilitate the entry of the iodinized contrast medium into the right coronary circulation by suspending the animals on their side on an acrylic board to achieve a frontal 2-ventricle view (Inagaki et al., 2021). Microangiograms (16-bit format, 33 fps) were recorded under steady-state conditions (sodium lactate 3 mL·h^−1^) at ~15 min after RV and arterial pressures stabilized to quantify RV perfusion. In study 1, at least 10 min was allowed between each treatment condition to ensure stable preparation and sufficient renal clearance of the contrast agent. At the conclusion of the experiment, the rats were killed by an overdose of 0.1 M KCl i.v. to arrest the heart in diastole.

### SAXS System and Protocol Study 3

The experiments were conducted at the public beamline BL40XU at SPring-8. Spiral-oriented fibers of the RV myocardial surface were perpendicularly aligned to the to the quasi-monochromatic X-ray beam (15 keV) with wavelength and dimensions as previously described (Figure 1A) (Pearson et al., 2007; Jenkins et al., 2013). For some of the rats, a superficial suture anchored to the LV apex was used to slightly rotate the heart, so that the RV surface was in the path of the X-ray beam (confirmed with a calibrated laser). The rats were positioned on a stage ~3 m away from the detector. SAXS patterns were then acquired at 10-ms intervals using an image intensifier and a fast CCD camera for a ~2s-period, sequentially repeated across the different layers of the RV wall. Diffraction patterns were digitally recorded using the HiPic 32 acquisition software. The rats were provided with a constant infusion of lactate (4 ml·h^−1^) to maintain stable blood and RV volumes throughout the protocol. At the conclusion of the experiment, the rats were killed by an overdose of 0.1M KCl as in study 1, and the SAXS patterns were acquired again to determine myosin mass transfer in the quiescent muscle (Jenkins et al., 2013). Offline analysis of the equatorial intensity ratio (I_1,0_/I_1,1_) and d_1,0_ spacing were performed as described earlier (Jenkins et al., 2013) for the epicardial, subepicardial, and subendocardial layers of the RV.

### Statistical Analyses

Data are presented as mean, with error bars representing SEM. A two-way analysis of variance was performed to determine treatment effects and/or differences in coronary vessel size class or myocardial layer. A one-way ANOVA was performed to determine the significance of differences among the drug treatment groups. Bonferroni's *post hoc* test was performed to establish between-group differences. A two-tailed *p* value of <0.05 was considered to be statistically significant.

## Results

### Acute Effects of Hexarelin on Rat Left Coronary Circulation (Study 1)

**Figure 2:**
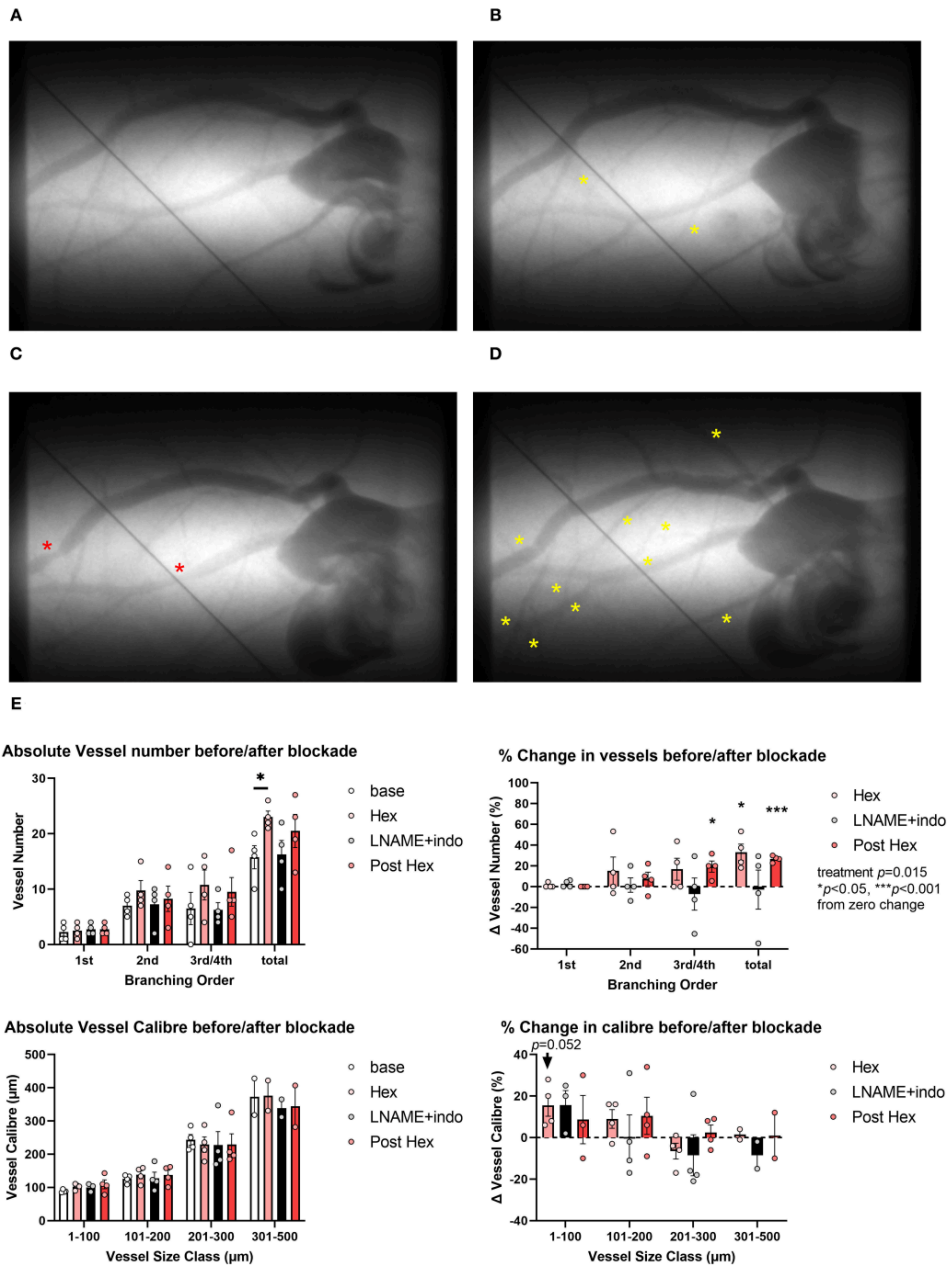
Representative examples of microangiograms acquired from a normal Sprague-Dawley rat during repeated imaging after hexarelin (hex) administration before and after blockade of nitric oxide synthase and cyclooxygenase. **(A)** Baseline image. **(B)** Ten minutes after hexarelin administration (100 μg·kg^−1^ intravenous, i.v.). **(C)** Twenty minutes after L-NAME (50 mg·kg^−1^ i.v.) and indomethacin (5 mg·kg^−1^) blockade. **(D)** Post blockade hexarelin administration. **(E)** Group mean visualized vessel number and vessel caliber and their relative changes from baseline (hexarelin, blockade) or relative to blockade (post hexarelin). Yellow asterisks indicate dilation or newly visualized segments. Red asterisks indicate constricted segments. Tungsten wire (50 μm) in the images was used for calibration. A two-way analysis of variance (ANOVA) was performed to compare factors of treatment group, vessel branching order, or size class followed by Bonferroni's *post hoc* test to establish within group differences. ^*^p < 0.05, ^***^p < 0.001 difference from zero change, one sample *t*-test.

The acute administration of hexarelin did not significantly alter mean arterial pressure or heart rate after 10 min (Supplementary Figure 1, protocol 1) but evoked modest increases in the number of contrast-perfused arterial vessel segments in the left coronary circulation of normal rats (no drug pretreatment) (Figures 2A,B,E). Hexarelin administration evoked a ~20–40% increase in visualized vessels, principally in the third and fourth order branching both before and after the inhibition of nitric oxide and prostanoid production, while blockade with L-NAME and indomethacin itself did not change the visible number of vessels (two-way ANOVA treatment *p* = 0.015). Change in vessel caliber of the arterial segments visible at baseline, following hexarelin administration before and after L-NAME and indomethacin blockade, was variable across all arterial vessel size classes (Figure 2E), but only increased in the smallest vessel size class prior to blockade (*p* = 0.052 relative to zero change). Nevertheless, focal constrictions observed in two of the four rats during blockade (Figure 2C) were ameliorated by subsequent hexarelin administration (Figure 2D). These preliminary findings suggest that in the normal heart, hexarelin increases coronary perfusion primarily through an increase in the perfusion of distal microvessels rather than an increase in radial dilation.

**Figure 3:**
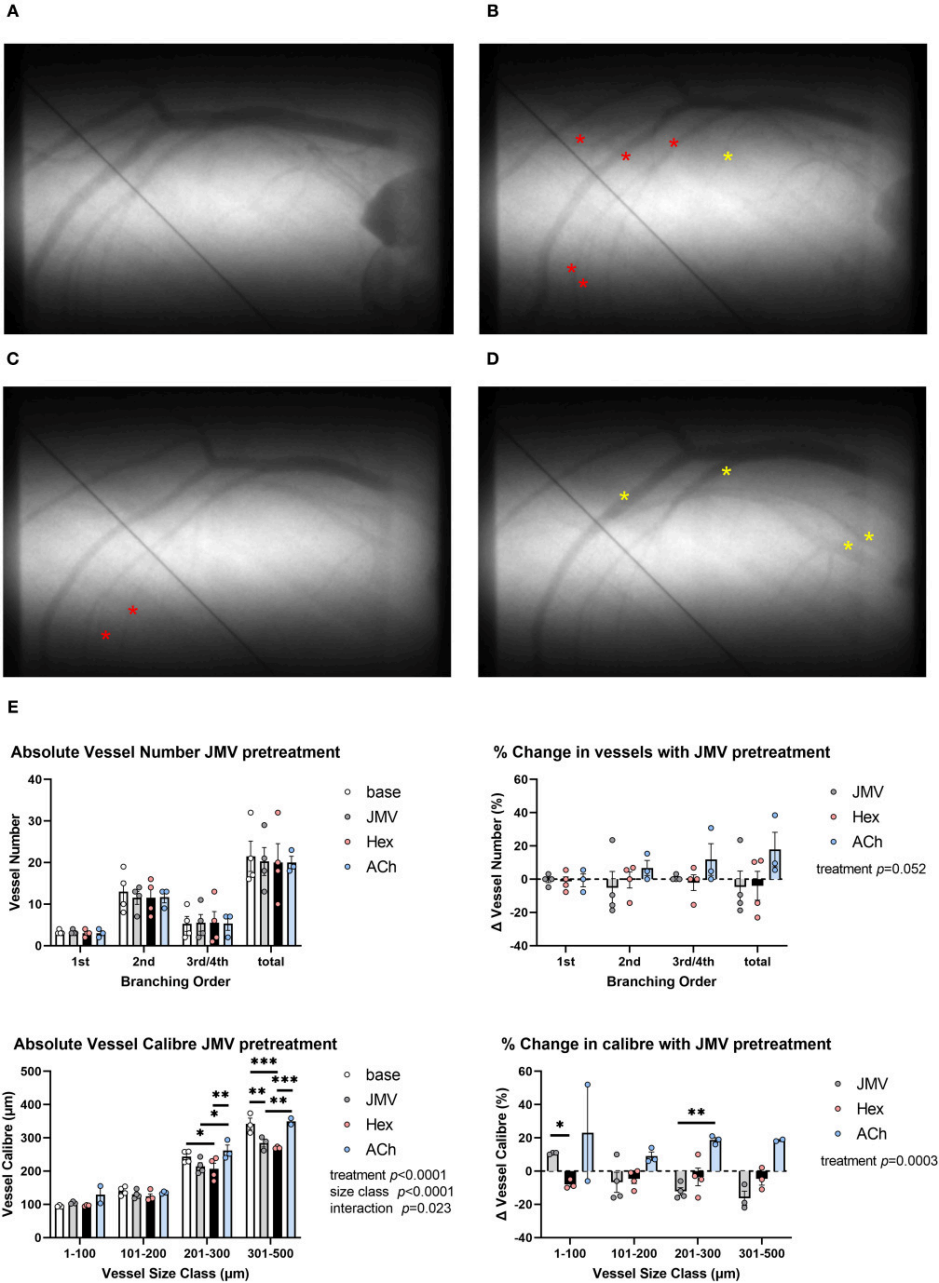
Representative examples of microangiograms acquired from a normal Sprague-Dawley rat during repeated imaging after hex administration following blockade of GHS-R1a with JMV2959 (JMV). **(A)** Baseline image. **(B)** Ten minutes after JMV2959 administration (5 mg·kg^−1^ i.v.). **(C)** Ten minutes after blockade hex administration (100 μg·kg^−1^). **(D)** ACh infusion (3 μg·kg^−1^·min^−1^). **(E)** Group mean visualized vessel number and vessel caliber and their relative changes from baseline (JMV blockade) or relative to blockade (hex, ACh). Yellow asterisks indicate dilation or new visual segments. Red asterisks indicate constricted segments. Tungsten wire (50 μm) in the images was used for calibration. A two-way ANOVA was performed to compare factors of treatment group, vessel branching order, or size class followed by Bonferroni's *post hoc* test to establish within group differences. ^*^p < 0.05, ^**^p < 0.01, ^***^p < 0.001 difference from zero change, one sample *t*-test.

To confirm whether the observed dilatory response to acute hexarelin administration was dependent on GHS-R1a, the response to hexarelin was examined in rats, following inhibition with JMV2959. Neither the baseline mean arterial pressure nor the heart rate differed between protocols 1 and 2. Ten minutes after JMV2959 administration, mean arterial pressure was not different from baseline; however, a variable but moderate decrease in HR was observed following hexarelin administration (Supplementary Figure 1, protocol 2), as opposed to a small but insignificant response to hexarelin in the absence of JMV2959 (protocol 1). Neither JMV2959 nor hexarelin administration post inhibition of GHS-R1a resulted in any significant change in visible vessel number (Figures 3A–E). On the other hand, in the presence of JMV2959 treatment, there was a strong trend for acetylcholine administration to evoke an increase in the number of perfused vessels (two-way ANOVA treatment *p* = 0.052). In contrast to visualized vessel number, drug treatments in protocol 2 had a significant effect on the change in caliber (two-way ANOVA treatment *p* = 0.0003) (Figure 3E). JMV2959 reduced the vessel caliber by up to 20% in the larger artery size classes relative to baseline, while subsequent hexarelin had no effect or evoked a minor constriction across the vascular network, as evident in Figure 3C. In these same rats, ACh induced an increase in vessel caliber, indicating that coronary vessels were capable of endothelium-dependent dilation. The preliminary findings of protocol 2 clearly showed that in the rat coronary circulation, at least, hexarelin-mediated vasodilation is dependent on GHS-R1a.

### General Animal Characteristics of the PH Model: Study 2

In comparison to the control rats, the SuHx rats were associated with a significantly lower body weight and significantly increased heart weight, RV weight, and RV weight to body weight ratio (Table 1), which are all consistent with a PH phenotype. In the SuHx rats, left lung wet weight also increased by ~25% compared to that in the control rats, although this difference did not reach statistical significance (Table 1). Hexarelin treatment had no significant effect on body weight, heart weight, left lung weight, or RV weight normalized to body weight relative to the SuHx vehicle rats (Table 1).

**Table 1:** General animal characteristics in study 2.

	**Control vehicle**	**SuHx3wk vehicle**	**SuHx3wk hexarelin**
Body weight (g)	474 ± 13	374 ± 14^***^	341 ± 19^****^
Heart weight (g)	1.19 ± 0.04	1.53 ± 0.11^*^	1.51 ± 0.06^*^
RV weight (g)	0.20 ± 0.01	0.46 ± 0.05^****^	0.43 ± 0.04^***^
Left lung weight (g)	0.56 ± 0.02	0.71 ± 0.06	0.72 ± 0.03
RV weight to body weight ratio (mg/g)	0.42 ± 0.03	1.26 ± 0.17^***^	1.30 ± 0.15^***^

*Data expressed as mean ± standard error of the mean (SEM)*.

* *p < 0.05*,

*** *p < 0.001, and*

**** *p < 0.0001 vs. control vehicle. RV, right ventricle; SuHx, sugen/chronic hypoxia*.

*Bold figures highlight significant differences*.

### Hemodynamics and Right Ventricular Functional Indices of the PH Model: Study 2

When compared to the control rats, none of the groups of SuHx rats exhibited a statistically significant difference in mean arterial pressure or heart rate under isoflurane anesthesia relative to the control group. As expected, RV end systolic pressure (ESP) was significantly greater in the SuHx group than in the control rats (*p* < 0.05, Table 2), which was not affected by hexarelin treatment (*p* < 0.01 vs. control, Table 2). The maximum rate of RV pressure development (dP/dt_max_) was significantly different in the hexarelin-treated SuHx rats relative to the controls (*p* < 0.05, Table 2). The contractility index (the quotient of the dP/dt_max_ and instantaneous pressure at the time point of dP/dt_max_) was nearly identical among all the groups (Table 2), suggesting that systolic function is maintained in the RV of the SuHx rats and was not altered by hexarelin treatment. RV end diastolic pressure (EDP) was not significantly elevated in the SuHx groups compared to the control rats, whereas the rate of RV pressure decay was significantly greater in both the SuHx vehicle-treated (*p* < 0.05 vs. control, Table 2) and SuHx hexarelin-treated (*p* < 0.01 vs. control, Table 2) rats.

**Table 2:** Right ventricular hemodynamic and function indices from study 2.

	**Control vehicle**	**SuHx3wk vehicle**	**SuHx3wk hexarelin**
**Hemodynamics**			
MAP	112 ± 6	114 ± 6	106 ± 9
Heart rate (BPM)	345 ± 15	302 ± 19	310 ± 18
**RV systolic indices**			
ESP (mmHg)	46 ± 3	88 ±9^*^	101 ±11^**^
dP/dt_max_(mmHg/s)	1,732 ± 191	3,064 ± 484	3,752 ±506^*^
Contractility Index (1/s)	64 ± 5	67 ± 5	69 ± 3
**RV diastolic indices**			
EDP (mmHg)	7.4 ± 0.7	10.2 ± 2.0	12.4 ± 1.8
dP/dt_min_ (mmHg/s)	−1,056 ± 123	−2,713 ±369^*^	−3,351 ±422^**^

*Data expressed as mean ± SEM. MAP, mean arterial pressure; ESP, end systolic pressure; dP/dt_max_, maximum rate of right ventricular pressure development; EDP, end diastolic pressure; dP/dt_min_, minimum rate of right ventricular pressure decay*.

* *p < 0.05*;

** *p < 0.01 vs. control vehicle*.

*Bold figures highlight significant differences*.

### Right Coronary Perfusion of the PH Model: Study 2

**Figure 4:**
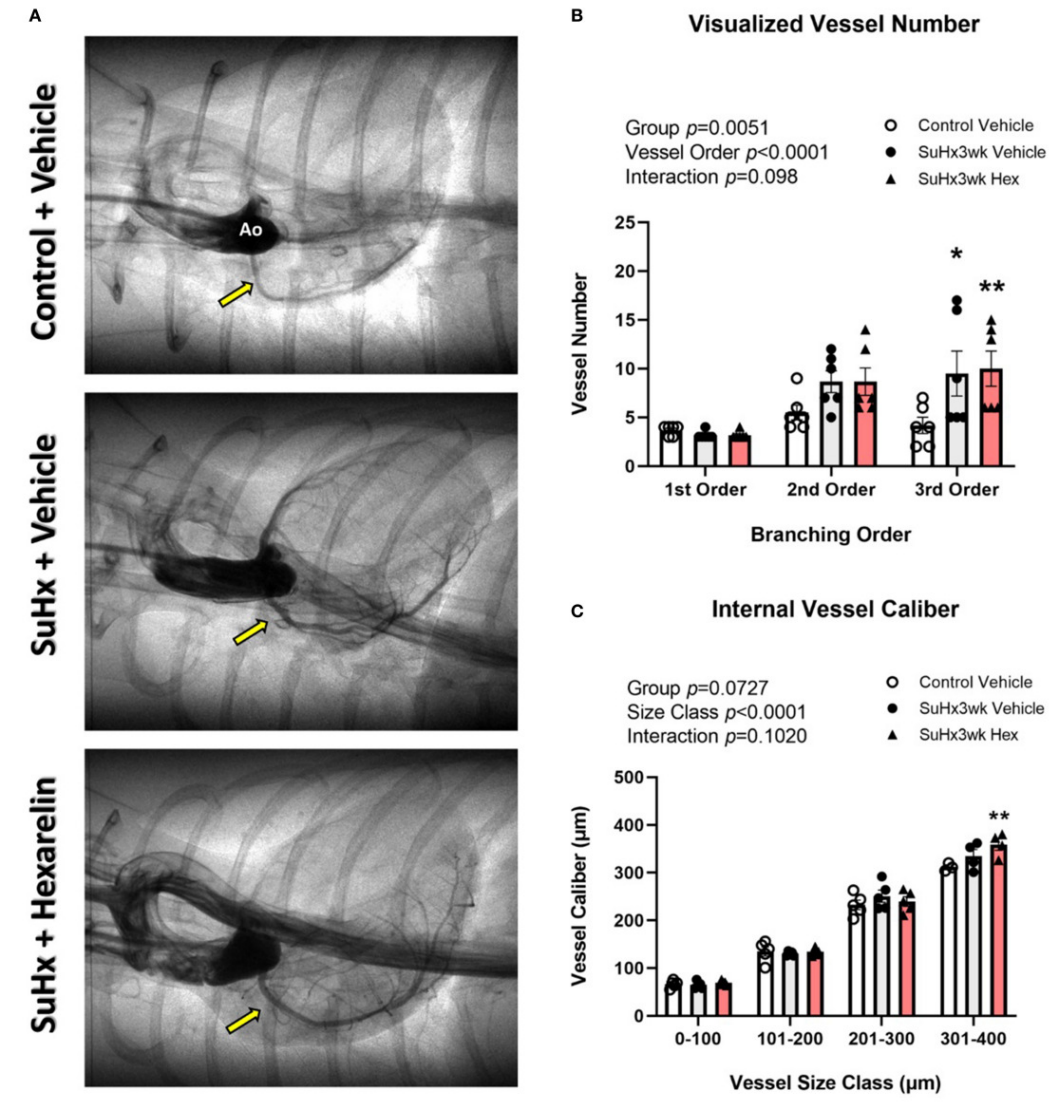
Assessment of right coronary perfusion in the control (*n* = 7), SuHx3wk vehicle (*n* = 7), and SuHx3wk hex (*n* = 7) rats. **(A)** Representative images of the right coronary circulation for the three treatment groups. **(B)** Visible vessel number across the right coronary vascular network as categorized by vessel branching order. When compared to the control vehicle rats, both the groups of SuHx3wk rats had a similar number of 1st order vessels, but second and third order vessel numbers were greater in the SuHx3wk rat groups than in the control vehicle rats. **(C)** Vessel caliber measurements. Vessel caliber was significantly different between the various vessel size classes (**C**, *p* < 0.0001), and significantly increased (*p* < 0.01) in the SuHx3wk hex-treated rats compared to control in the 301–400 μm vessel size class **(C)**; however, no other significant changes were observed between the groups at any other vessel size class. A two-way ANOVA was performed to compare differences followed by Bonferroni's *post hoc* test to establish within group differences. ^*^p < 0.05, ^**^p < 0.01 vs control vehicle. Data are expressed as mean ± standard error of the mean (SEM).

The SuHx rats did not show any difference in the total visualized arterial vessel segment number compared to the control rats (Figure 4A). However, when vessel number was examined by branching order, significantly more third order vessels were observed in both SuHx groups compared to the vehicle-treated control rats (SuHx 3wk vehicle, *p* < 0.05 and SuHx 3wk hexarelin, *p* < 0.01; Figure 4B). Within group comparisons revealed that vessels in the size class 301–400 μm were significantly larger in the hexarelin-treated SuHx rats when compared to control (*p* < 0.01, Figure 4C). Hence, these early findings from SR microangiography suggest that hexarelin did not notably alter steady-state coronary perfusion in the SuHx model, but it remains to be determined if chronic hexarelin treatment can prevent the onset of pronounced endothelial dysfunction in the RCA.

### SAXS Experiments: Study 3

**Figure 5:**
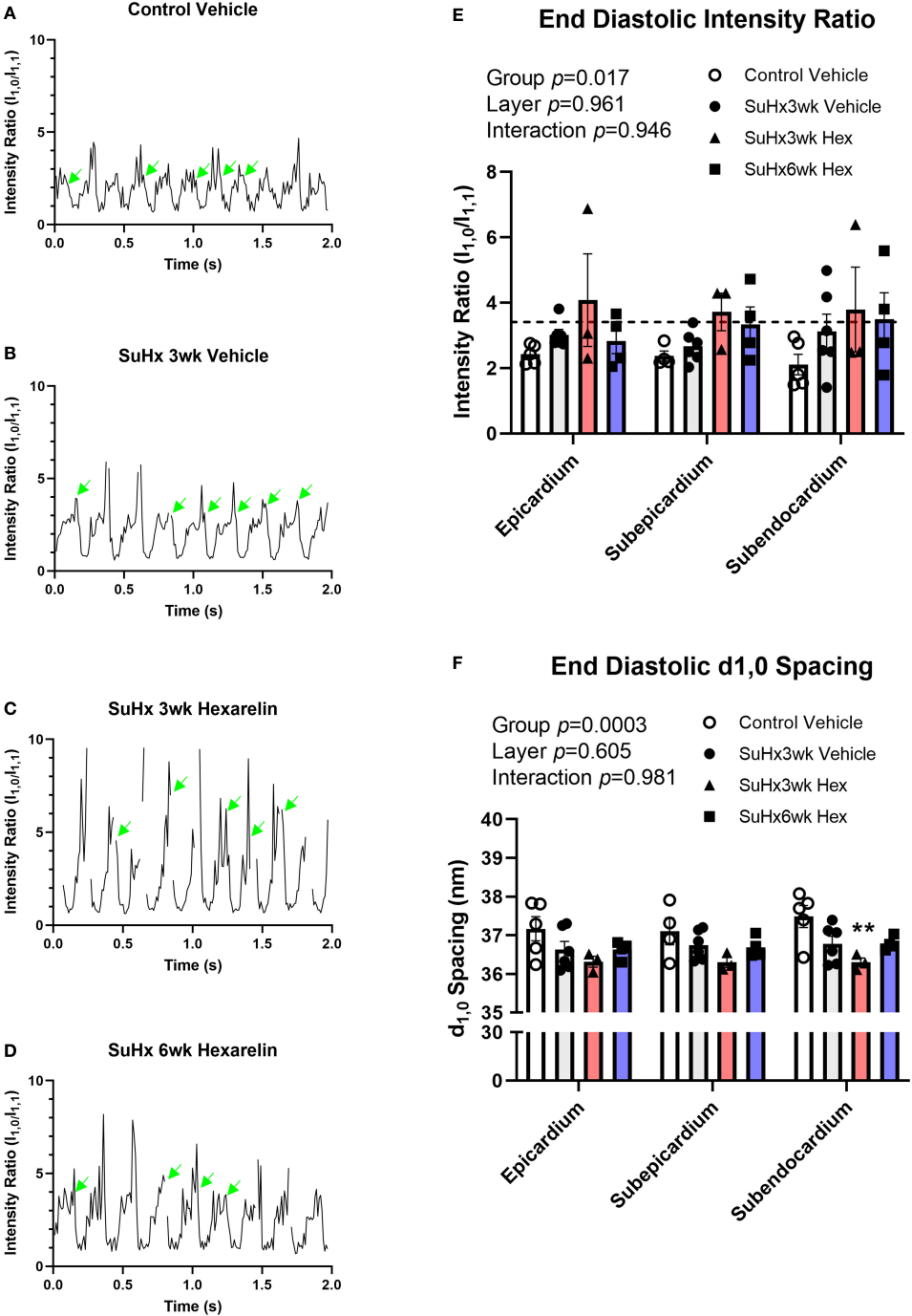
Cyclic changes in the SAXS intensity ratio over several cardiac cycles for **(A)** control, **(B)** SuHx3wk vehicle, **(C)** SuHx3wk hex, and **(D)** SuHx6wk hex. Green arrows indicate the time points where the end diastolic (ED) intensity ratio could be established. Gaps indicate time points where artifacts prevented the analysis of SAXS patterns. The significant differences between the groups were established for the ED intensity ratio (**E**, *p* = 0.017), with differences in ED intensity ratio becoming most pronounced in the deeper subendocardial layer when comparing the control vehicle rats with the SuHx3wk vehicle, SuHx3wk hex, and SuHx6wk hex rats. The broken line in **(E)** represents the mean intensity ratio for all the rats during muscle quiescence after the administration of 0.1 M KCl (i.v.). Myosin interfilament (d_1,0_) differed significantly between the groups (**F**, *p* = 0.0003), with interfilament spacing being smaller on the whole in all the groups of SuHx rats compared to the control vehicle rats. The interfilament spacing was significantly smaller in the SuHx3wk hex group than in the control vehicle (**F**, *p* < 0.01) in the subendocardium. The interfilament spacing tended to increase in the SuHx6wk hex group when compared to SuHx3wk hex in all myocardial layers **(F)**. A two-way ANOVA was performed to compare differences followed by Bonferroni's *post hoc* test to establish within group differences. ^**^p < 0.01 vs. control vehicle. Data are expressed as mean ± SEM.

The cyclical changes in SAXS intensity ratio were consistent with the cardiac cycle across all the groups due to myosin mass transfer to the actin thin filaments (Figures 5A–D), as we have previously demonstrated in the LV. Focusing on diastolic function, there were significant differences among the groups in the end diastolic (ED) intensity ratio (2-way ANOVA group *p* = 0.017). In the control rats, ED intensity ratio did not differ with respect to myocardial layer (Figure 5E), nor did the ED intensity ratio exceed that of the intensity ratio of the quiescent state (broken line, Figure 5E). This quiescent state intensity ratio did not differ among the groups (mean 3.41 ± 0.17, *n* = 3–7/group) and was similar to earlier reports for normal LV myocardium (Jenkins et al., 2013; Waddingham et al., 2015). The differences in ED intensity ratio among the groups were primarily due to increased myosin head displacement toward the actin filaments (ED intensity ratio mean 2.1–2.4) in the control vehicle-treated rats, while the SuHx rats showed ED intensity ratios similar to the quiescent state (Figure 5E). Moreover, treating the SuHx rats with hexarelin for an extended period of 6 weeks post hypoxia (SuHx6wk hexarelin) did not significantly change diastolic mass transfer relative to the vehicle and to the SuHx3wk hexarelin groups (Figure 5E).

Myosin interfilament (d_1,0_) spacing did not differ significantly between myocardial layers in any of the groups, but the treatment group means differed significantly (two-way ANOVA group *p* = 0.0003, Figure 5F). Interfilament spacing in the hexarelin-treated SuHx 3wk rats was ~1 nm smaller than in the control rats (Figure 5F), reaching statistical significance in the subendocardial layer (*p* < 0.01 vs. control vehicle, Figure 5F). There were no differences in interfilament spacing in the hexarelin-treated SuHx 6wk group when compared to any other group in the different myocardial layers (based on pairwise comparisons). From a biomechanical perspective, the smaller interfilament spacing in hexarelin-treated SuHx rats at 3-weeks relative to the control rats implies that cardiac fibers in the former were operating at a longer sarcomere length relative to the latter.

## Discussion

In this study, we provide direct evidence that hexarelin act as a vasodilator in the healthy rat coronary microcirculation, and that this involved activation of the GHS-R1a receptor pathway as reported for ghrelin (Pearson et al., 2019). Furthermore, for the first time in a PH model, we show that: (1) more myosin heads appeared to become super-relaxed, with displacement away from actin binding sites, while (2) myosin interfilament spacing at diastole was additively reduced by the combination of PH and chronic hexarelin treatment. However, we must acknowledge that this is an ongoing study with a somewhat limited number of animals reported, and as such, further confirmation and more detailed studies are required focusing on both acute and chronic effects of hexarelin treatment in healthy and disease states other than myocardial infarction.

To date, there have been few investigations of the vascular effects of hexarelin, other than showing that it reduces apoptosis in ischemia (Mao et al., 2014a). Hexarelin-mediated dilation is shown in this study to be dependent on local GHS-R1a, not because of change in perfusion pressure, as mean arterial pressure did not change significantly after acute administration. Our novel results suggest that in the rat circulation, hexarelin-mediated dilation is in part due to nitric oxide release and partly due to endothelium-derived hyperpolarization, since the residual vasodilation that resulted in an increase in distal perfusion following L-NAME and indomethacin blockade is as large as the dilation prior to blockade. However, our data also suggest that the hexarelin-mediated dilation in both the rat and mouse coronary circulations is not due to nitric oxide facilitating endothelium-derived hyperpolarization, as the combination of indomethacin with apamin and charybdotoxin infusion did not prevent the increase in microvessel perfusion to hexarelin (Supplementary Figures 2, 3). Nonetheless, these early findings suggest that there might be species differences in the contribution of nitric oxide and endothelium-derived hyperpolarization to hexarelin-mediated vasodilation, which needs further investigation.

Pulmonary hypertension remains an incurable cardiovascular disease for which currently recommended vasodilator therapies, at best, slow the progression of disease (Thenappan et al., 2018; Prisco et al., 2020). Moreover, there is no specific therapy targeted to RV failure in PH. To demonstrate the utility of SR imaging approaches, which complement state-of-the-art cardiology techniques for such studies on the pathogenesis and prevention of PH, we briefly described herein how RV function is affected by chronic administration of a GHS peptide that is widely considered as a promising therapy to reduce neurohormonal activation and adverse remodeling in cardiovascular disease (Berti et al., 1998; Filigheddu et al., 2001; Mao et al., 2013a, 2014a,b; Mcdonald et al., 2020). The novel and somewhat surprising findings in this study, even with limited sample sizes, are that chronic simultaneous administration of hexarelin with SuHx treatment to induce severe PH did not prevent the pathogenesis of RV hypertrophy or RV myocardial dysfunction in the young adult male rats. Whether chronic hexarelin treatment can ameliorate or prevent the right coronary endothelial dysfunction, which is a characteristic of severe PH (Inagaki et al., 2021), needs to be investigated in future studies. Below we focus on the significance of our *in vivo* observations to better understand cardiomyocyte dysfunction within the RV in a complex multiorgan disease state such as PH.

Myosin super relaxation is increasingly recognized as either a state of energy conservation or deficit where myosin heads are unavailable for weak or strong-binding cross-bridge states with actin, whereby myosin heads remain in proximity to the myosin thick filament backbone rather than the actin filaments (Toepfer et al., 2020; Schmid and Toepfer, 2021). In comparison to the control vehicle rats, we observed that more myosin heads are displaced from the actin filaments at end diastole (based on SAXS intensity ratio) in the SuHx rat hearts. Previously, we have established that a similar phenomenon is observed in type 1 and 2 diabetes mellitus models in early stages of diastolic dysfunction due to alterations in the phosphorylation state of myosin myofilament proteins (Waddingham et al., 2015, 2019). In brief, various studies provide evidence that altered protein kinase activity and phosphorylation states of myosin-binding protein and or myosin light chain 2 directly determine myosin head proximity to actin filaments and thereby cardiomyocyte and ventricular relaxation rates in prediabetes, hypertrophic cardiomyopathy, and heart failure (Colson et al., 2008; Tong et al., 2008; Waddingham et al., 2019). Changes in protein kinase activity and myofilament phosphorylation state, indeed most posttranslation modifications of sarcomeric proteins in PH remain poorly understood in relation to RV myocardial and cardiomyocyte contractile function. Others have shown that ghrelin applied to the RV papillary muscles from control and monocrotaline PH model rats dose-dependently reduced inotropy and relaxation rates to a similar extent (Soares et al., 2006). However, in this study, the hexarelin-treated SuHx rats did not show enhanced impairment of RV relaxation (dP/dt_min_) relative to the vehicle-treated rats. Controversially, some studies suggested that both ghrelin and hexarelin have positive inotropic effects on cardiomyocytes (Sun et al., 2010) and on healthy volunteers (Bisi et al., 1999). In this initial study, we did not establish how cross-bridge dynamics in systole and diastole are affected by β-adrenoceptor activation during a cardiac stress test (e.g., dobutamine infusion), which is challenging under anesthetized conditions in this model. Therefore, at present, we do not know if the speculated diastolic myosin super relaxation state *in vivo* is altered by changes in protein kinase phosphorylation of the myofilaments. Since increasing evidence indicates that transmural gradients in sarcomeric contractile-relaxation function and protein kinase activities exist in the LV (Van Der Velden et al., 2011; Haynes et al., 2014; Pan et al., 2018) and presumably in the hypertrophied RV wall, it would be informative to investigate how regional enzyme activities relate to contraction-relaxation properties of the same muscle fibers in the RV in PH.

Length-dependent activation of cardiac muscle is an important mechanism that facilitates an increase in the force of contraction when venous return and preload are increased on a beat-to-beat basis. While the complex interactions between cardiac troponins, myosin proteins, and titin have been shown to be critical to length-dependent activation or the Frank-Starling mechanism, many details of this mechanism remain unresolved (Fukuda et al., 2001, 2010; Caremani et al., 2018; Li et al., 2018). Intact cardiac fibers maintain an inverse relationship between myosin interfilament spacing and sarcomere length as a consequence of constant cell volume behavior and limitations of a cell membrane (Yagi et al., 2004), whereby cell shortening causes myosin lattice spacing increase and, conversely, stretch of cardiac sarcomeres reduces interfilament spacing and increases the probability of myosin cross-bridge formation in systole (Pearson et al., 2007). In this study the observation that hexarelin enhanced the reduction in diastolic myosin interfilament spacing suggests that the hexarelin-treated SuHx rats are likely to be operating at longer sarcomere lengths across the RV transmural wall. We estimate that the sarcomere, length based on the published correlation with d_1,0_ spacing reported for intact rat cardiac fibers (Yagi et al., 2004) within the subendocardium, was 2.1 ± 0.03, 2.18 ± 0.02 nm, 2.24 ± 0.01, and 2.18 ± 0.01 nm in the control vehicle, SuHx3wk vehicle, SuHx3wk hexarelin, and 6wk hexarelin groups, respectively. While it is possible, but very difficult to establish epicardial sarcomere length *in situ* with laser diffraction, the extrapolations from isolated intact fibers are comparable to that now obtained with fluorescence nanoimaging of transfected fluorophore proteins (Kobirumaki-Shimozawa et al., 2015). On the basis of the observed differences in myosin interfilament spacing, it is not possible to establish whether the RV cardiac fibers might be stretched beyond the optimal sarcomere length for force development in the hexarelin-treated SuHx rats without further evidence, for example, cardiac magnetic resonance imaging or micro-computed tomography-derived RV volume determinations to demonstrate evidence of fiber stretching associated with volume overload. Nonetheless, we can speculate that the modest increases in cardiac fiber stretching contributed to length-dependent activation and has sustained RV contractility at least in the early phase of PH in the SuHx model, as d_1,0_ spacing was reduced relative to the control in all the SuHx groups. Further investigations of cardiac muscle biomechanics and inotropy in the *in situ* RV and isolated trabeculae or cardiomyocytes in combination with characterization of protein kinase and phosphoprotein changes in the RV are needed to understand the pathogenesis of RV failure, the effects of chronic hexarelin on sarcomere function and cardiac remodeling.

Interestingly, the synchrotron-based imaging approaches in combination with basic morphometric data for the rat hearts suggest that the hexarelin GHS, at least at the dose investigated, was not able to prevent the neurohormonal activation and subsequent RV hypertrophy at the onset of severe PH. However, we and other researchers have shown ghrelin to be effective in preventing the pathogenesis of PH in terms of ameliorating pulmonary vessel remodeling, RV hypertrophy and RV dysfunction in chronic hypoxia and monocrotaline-treated rats during the compensatory phase of PH (Henriques-Coelho et al., 2004; Schwenke et al., 2008b, 2011). In this study, as pulmonary vascular function was not assessed with SR microangiography, we cannot exclude the possibility that pulmonary vascular dysfunction might be partly ameliorated by chronic hexarelin treatment. However, the fact that systolic peak RV pressure and end-diastolic RV pressure were not reduced in the hexarelin-treated SuHx rats compared to the vehicle-treated ones suggests that this is probably unlikely, as this implies that pulmonary vascular resistance must be greatly elevated relative to the control rats. Assessment of gene-protein expression changes in relation to GHS signaling and neurohormonal mechanisms in the lungs and RV is required to understand these findings, which is beyond the scope of this article focusing on bridging techniques in cardiovascular physiology.

## Conclusions

The combination of SR-based imaging techniques utilized in this study allowed for the investigation of microlevel regulation of *in situ* cardiomyocyte function and microvessel perfusion in the early stages of cardiovascular disease in preclinical models and can, therefore, add further knowledge to studies with complementary conventional macrolevel cardiovascular techniques. Third- and fourth-generation SR research facilities are available worldwide with merit-based access systems that are capable of performing the *in situ* experiments as described here. Indeed, SAXS experiments on *ex vivo* muscle preparations are routinely performed in many facilities. These approaches we believe can provide new insights into cardiovascular disease at the nano- and microlevels.

## Data Availability Statement

The raw data supporting the conclusions of this article will be made available by the authors, without undue reservation.

## Ethics Statement

The animal study was reviewed and approved by the Animal Experimentation Committee of the National Cerebral and Cardiovascular Center and the Japan Synchrotron Radiation Research Institute.

## Author Contributions

MW, MS, TO, and JP conceived and designed the experiments and interpreted the data. MW, HT, TS, RA, HJ, CO, DS, RK, and JP performed the experiments. KA, KUm, MH, and KUe provided technical expertise and support at SPring-8. MW and JP analyzed the data and wrote the manuscript. All authors read and approved the final version of the manuscript.

## Funding

This study was supported by the JSPS KAKENHI (Grant Nos: 19H03405 and 19K16498) and an Investigator Initiated Study grant from Janssen Pharmaceuticals and Nippon Shinyaku (awarded to TO).

## Conflict of Interest

MW, RA, and TO belong to a department endowed by Nippon Shinyaku Co. The remaining authors declare that the research was conducted in the absence of any commercial or financial relationships that could be construed as a potential conflict of interest.

## Publisher's Note

All claims expressed in this article are solely those of the authors and do not necessarily represent those of their affiliated organizations, or those of the publisher, the editors and the reviewers. Any product that may be evaluated in this article, or claim that may be made by its manufacturer, is not guaranteed or endorsed by the publisher.
