# Supplementary material for: Using Synchrotron Radiation Imaging Techniques to Elucidate the Actions of Hexarelin in the Heart of Small Animal Models
Source: Front Physiol. 2022 Jan 21;12:766818. doi: 10.3389/fphys.2021.766818 (PMC8814524; doi:10.3389/fphys.2021.766818)
Supplement: Supplementary file 1 [file Data_Sheet_1.PDF]

Using synchrotron radiation imaging techniques to elucidate the actions of hexarelin in the heart in small animal models

Mark T Waddingham[1], Hirotsugu Tsuchimochi[2], Takashi Sonobe[2], Ryotaro Asano[1], Huiling Jin[2], Connie PC Ow[2], Daryl O Schwenke[3], Rajesh Katare[3], Kohki Aoyama[4], Keiji Umetani[4], Masato Hoshino[4], Kentaro Uesugi[4], Mikiyasu Shirai[1,2], Takeshi Ogo[1†], James T Pearson[2,5†]

[1]Department of Advanced Medical Research for Pulmonary Hypertension, National Cerebral and Cardiovascular Center, Suita, Osaka, Japan

[2]Department of Cardiac Physiology, National Cerebral and Cardiovascular Center Research Institute, Suita, Osaka, Japan

[3]Department of Physiology, School of Biomedical Sciences, HeartOtago, University of Otago, Dunedin, New Zealand

[4]Japan Synchrotron Radiation Research Institute, Harima, Hyogo, Japan

[5]Monash Biomedicine Discovery Institute and Department of Physiology, Monash University, Clayton, VIC, Australia

Corresponding Author:

Professor James T Pearson PhD

Department of Cardiac Physiology

National Cerebral and Cardiovascular Center Research Institute

6-1 Kishibeshinmachi, Suita-shi

564-8565 Osaka

Japan

Email: jpearson@ncvc.go.jp

† Authors contributed equally and share senior authorship

Supplement Methods

Surgical Preparation – Coronary Microangiography (Study 1 and 2)

Rats were anaesthetised with isoflurane (4% for induction, 1.5-2% for maintenance mixed 1:1 in 100% O_2) and once deep surgical anaesthesia was confirmed by the absence of the pedal reflex, were tracheotomised and mechanically ventilated (~80-85 breaths·min^{-1}). Body temperature was maintained at 37°C throughout the protocol with the use of a rectal thermistor coupled to a thermostatically controlled heat pad. In study 1 a femoral artery and the right jugular vein were cannulated for the continuous monitoring of systemic arterial pressure and for fluid replacement/drug delivery, respectively. The right carotid artery was isolated and a 20-gauge radiopaque catheter (Argyle) was inserted and advanced to the entrance of the aortic valve with the bevel tip facing the left coronary artery. In study 2 (PH model)3 the femoral artery and vein were cannulated for the continuous monitoring of systemic arterial pressure and for fluid replacement/drug delivery, respectively. The right external jugular vein was isolated and an 18-gauge BD Angiocath catheter (Becton Dickinson) with the tip bent at a 30° angle was inserted and advanced into the RV for the measurement of pressure and evaluation of RV haemodynamics. A 20-gauge radiopaque catheter (Argyle, Nihon Covidien, Tokyo, Japan) was then inserted into the right common carotid artery and positioned so that the catheter's tip was at the entrance of the aortic valve with the bevel tip facing the RCA.

Surgical Preparation – Small Angle X-Ray Scattering (SAXS) (Study 3)

Rats were anaesthetised, tracheotomised, ventilated and warmed as per Study 1. A thoracotomy was then performed to allow an unobstructed path for the X-ray beam to the heart, as described previously (Jenkins et al., 2013; Waddingham et al., 2019). The right common carotid artery and right jugular vein was then isolated and cannulated for monitoring of blood pressure and fluid delivery, respectively. The heart was then coated in liquid paraffin oil and lungs covered in Ringer's lactate-soaked gauze to prevent drying.

Jenkins, M.J., Pearson, J.T., Schwenke, D.O., Edgley, A.J., Sonobe, T., Fujii, Y., Ishibashi-Ueda, H., Kelly, D.J., Yagi, N., and Shirai, M. (2013). Myosin heads are displaced from actin filaments in the in situ beating rat heart in early diabetes. *Biophysical journal* 104**,** 1065-1072. doi:10.1016/j.bpj.2013.01.037

Waddingham, M.T., Sonobe, T., Tsuchimochi, H., Edgley, A.J., Sukumaran, V., Chen, Y.C., Hansra, S.S., Schwenke, D.O., Umetani, K., Aoyama, K., Yagi, N., Kelly, D.J., Gaderi, S., Herwig, M., Kolijn, D., Mugge, A., Paulus, W.J., Ogo, T., Shirai, M., Hamdani, N., and Pearson, J.T. (2019). Diastolic dysfunction is initiated by cardiomyocyte impairment ahead of endothelial dysfunction due to increased oxidative stress and inflammation in an experimental prediabetes model. *J Mol Cell Cardiol* 137**,** 119-131. doi:10.1016/j.yjmcc.2019.10.005

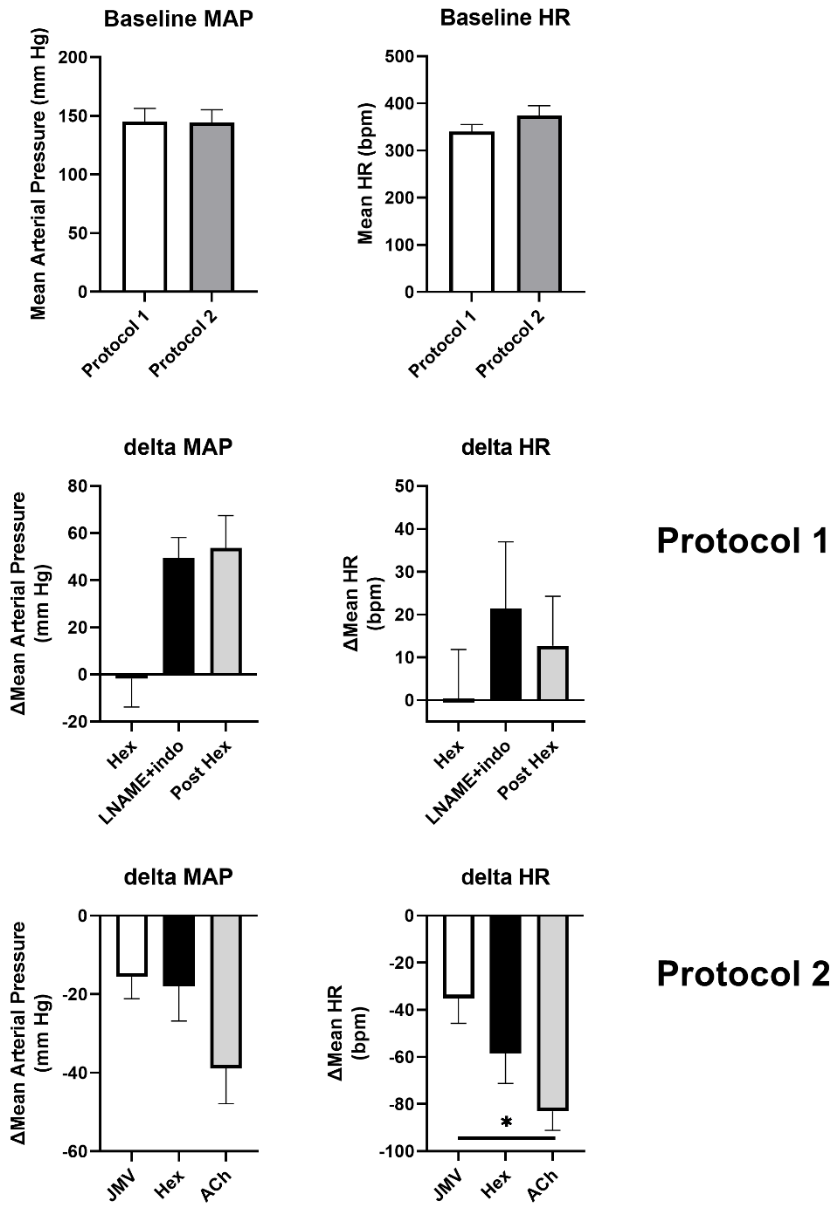

Supplementary Figure 1. Haemodynamic changes during protocols 1 and 2 to assess the acute effects of hexarelin in the adult rat coronary circulation. In both protocols baseline mean arterial pressure (MAP) and mean heart rate (HR) were similar. During protocol 1 hexarelin (hex) administration did not significantly MAP or mean HR before inhibition of nitric oxide synthase (L-NAME) and cyclooxygenase (Indomethacin, Indo) or after inhibition (changes relative to LNAME+indo). During protocol 2, pretreatment with GHS-R1a antagonist (JMV2959 6mg·kg^{-1} i.p.) resulted in minor decreases in MAP and HR that were not significantly altered by hexarelin. Final ACh infusion evoked a further small decrease in MAP and significant decrease in mean HR relative to JMV administration. A one-way ANOVA was used to compare differences followed by a Bonferroni's *post hoc* test to establish between group differences. **p*<0.05 vs. JMV. Data are expressed as mean ± SEM.

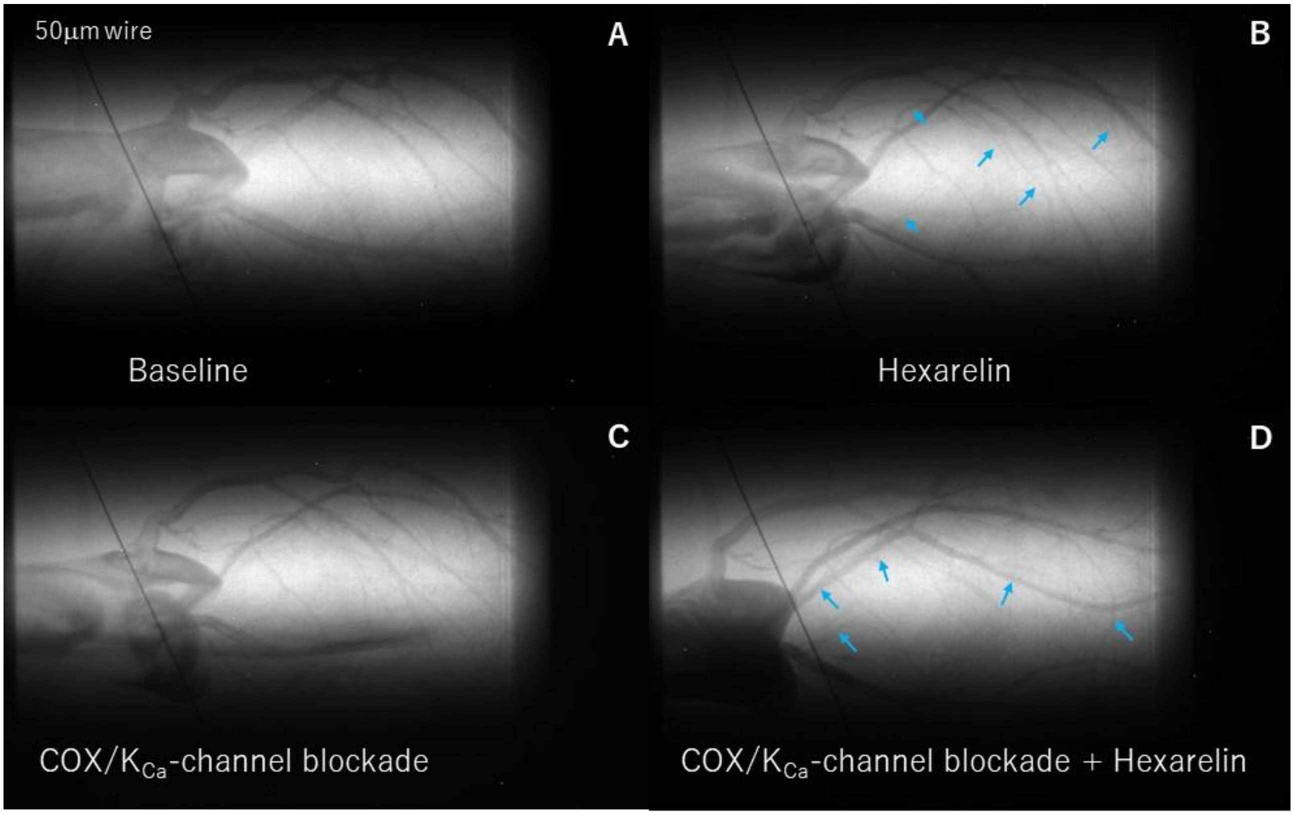

Supplementary Figure 2. A representative example of hexarelin mediated vasodilation in a male Sprague-Dawley rat coronary circulation before and after inhibition of cyclooxygenase (COX) and calcium-activated potassium channels (K_{Ca}). (**A**) Baseline image. (**B**) 10 min after hexarelin administration (100µg·kg^{-1} i.v.) shows dilation and or new vessels indicated by blue arrows. (**C**) 10min after inhibition of COX (indomethacin 5mg/kg i.v.) during K_{Ca} blockade (Apamin 0.5µM and Charybdotoxin 0.05µM i.v.). (**D**) repeat administration of hexarelin evoked dilation is well maintained. Surgical preparation and image acquisition as described in study 1.

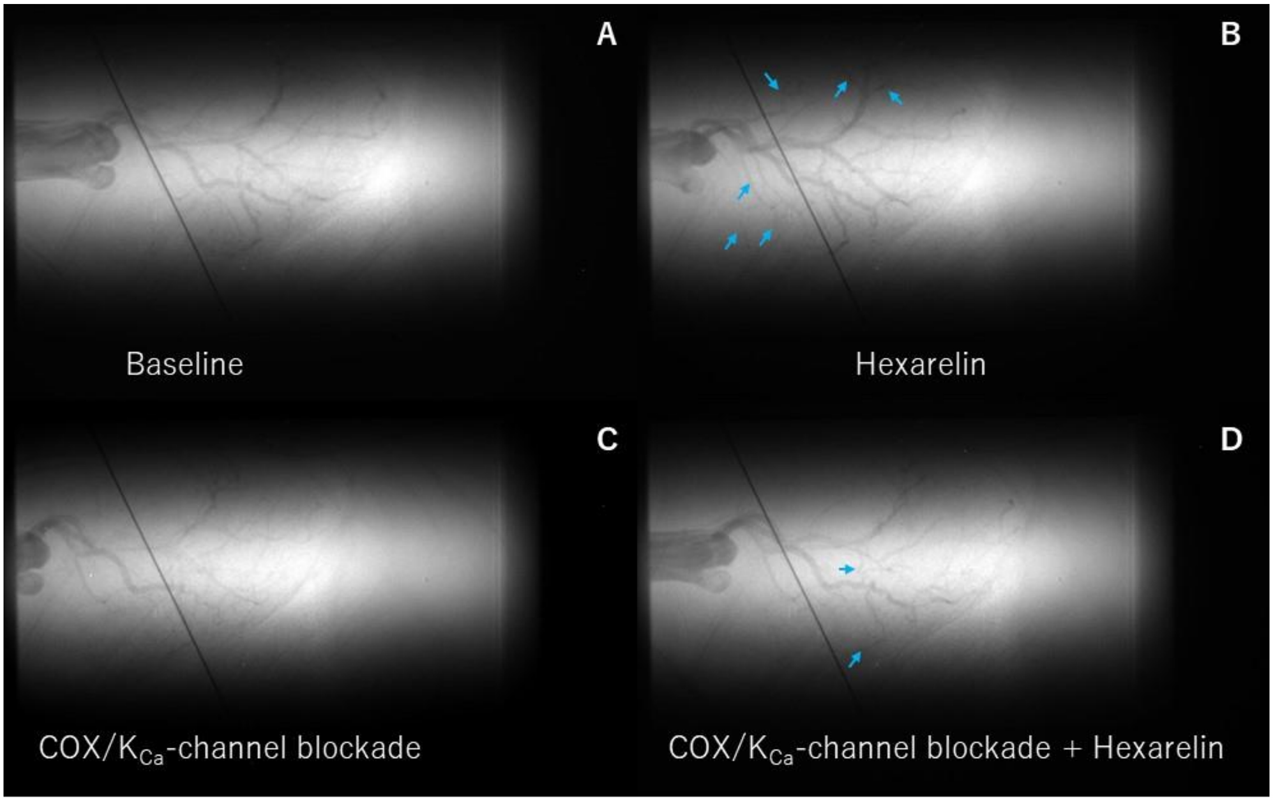

Supplementary Figure 3. A representative example of hexarelin mediated vasodilation in a male C57Bl6/J mouse coronary circulation before and after inhibition of cyclooxygenase (COX) and calcium-activated potassium channels (K_{Ca}). (**A**) Baseline image. (**B**) 10 min after hexarelin administration (100µg·kg^{-1} i.v.) shows dilation and or new vessels indicated by blue arrows. (**C**) 10min after inhibition of COX (indomethacin 5mg/kg i.v.) during K_{Ca} blockade (Apamin 0.5µM and Charybdotoxin 0.05µM i.v.). (**D**) repeat administration of hexarelin evoked modest dilation relative to administration before blockade, suggesting that endothelium-derived hyperpolarisation has a large contribution to hexarelin mediated dilation than nitric oxide. Surgical preparation and image acquisition as described in study 1.
